# Encapsulation of *Lactobacillus fermentum* K73 by Refractance Window drying

**DOI:** 10.1038/s41598-019-42016-0

**Published:** 2019-04-04

**Authors:** Stephania Aragón-Rojas, María Ximena Quintanilla-Carvajal, Humberto Hernández-Sánchez, Alan Javier Hernández-Álvarez, Fabian Leonardo Moreno

**Affiliations:** 1 Universidad de La Sabana, Faculty of Engineering. Doctorado en Biociencias. Campus Universitario del Puente del Común, Km 7 Autopista Norte de Bogotá, Chía, Cundinamarca Colombia; 2 Universidad de La Sabana, Faculty of Engineering. Grupo de Investigación en Procesos Agroindustriales Campus Universitario del Puente del Común, Km 7 Autopista Norte de Bogotá, Chía, Cundinamarca Colombia; 3 Escuela Nacional de Ciencias Biológicas, Instituto Politécnico Nacional. Av. Wilfrido Massieu esq. Cda. M. Stampa, UP Adolfo López Mateos, Ciudad de México, 07738 Mexico; 4 Food Research and Development Centre, 3600 boulevard Casavant, Saint-Hyacinthe, J2S 8E3 Quebec Canada

## Abstract

The purpose of this work was to model the survival of the microorganism and the kinetics of drying during the encapsulation of *Lactobacillus fermentum* K73 by Refractance Window drying. A whey culture medium with and without addition of maltodextrin were used as encapsulation matrices. The microorganism with the encapsulation matrices was dried at three water temperatures (333, 343 and 353 K) until reaching balanced moisture. Microorganism survival and thin layer drying kinetics were studied by using mathematical models. Results showed that modified Gompertz model and Midilli model described the survival of the microorganism and the drying kinetics, respectively. The most favorable process conditions found with the mathematical modelling were a drying time of 2460 s, at a temperature of 353 K. At these conditions, a product with 9.1 Log CFU/g and a final humidity of 10% [wet basis] using the culture medium as encapsulation matrix was obtained. The result shows that Refractance Window can be applied to encapsulate the microorganism probiotic with a proper survival of the microorganism.

## Introduction

Probiotics have been defined by the FAO/WHO as “live microorganisms which when administered in adequate amounts confer a health benefit on the host”^1^. Evidence of the effect of probiotics on consumer health^2–4^ has driven the development of strategies to include them in food matrices and to generate non-traditional dairy functional foods, such as Oaxaca cheese^5^ or ice cream^6^ and non-dairy products such as bread^7^, fermented sausages^8^, carrot juice^9^, among others; these food products represent 60–70% of the functional food market^10^, this being an opportunity for the development of new products.

Until now, different species of probiotic microorganisms have been selected according to their characteristics. Strains such as *Lactobacillus fermentum* K73, isolated from *suero costeño* (typical fermented food from the Colombian Atlantic coast) have shown to have a hypocholesterolemic effect to adsorb cholesterol on its cell membrane and for the activity of the bile salt hydrolase enzyme^11^. Due to the studied potential of this strain, it is possible to include it in a functional food.

Functional foods enriched with probiotic microorganisms should declare a minimum concentration of 10^6^ per gram or milliliter at the time of consumption^12^. Encapsulation is an alternative to improve the probiotics survival during its inclusion in food, storage and to protect it from gastrointestinal stress; it has been defined as a technology for packaging a bioactive compound that can be in a solid, liquid or gaseous state within a matrix^13^.

Different matrices to encapsulate probiotics have been used to preserve its functionality sush as: whey proteins^14^, maltodextrin^15^, gum arabic^16^, among others. Whey proteins have been studied in the food industry due to their structural and physicochemical properties and its acting as a “natural delivery system” at the gastrointestinal tract level^17,18^.

The encapsulation of probiotics has been done through the use of emulsions^19^, extrusions^20^ and through the use of different drying technologies^21^; the selection of these techniques depends on the food product where the probiotic will be added. Additionally, the technique must guarantee the survival of the probiotic and a low moisture content for the stability of the product^22^. An alternative in drying technologies that has not yet been explored for probiotic encapsulation is the Refractance Window drying (RW). The RW is a technique used for concentrating and drying solutions and purées that allows obtaining a product in the form of a flake or a film^23^. In the RW drying, the solution or purée is placed on a transparent polyester film, known as Mylar® (DuPont Polyester Film Enterprise, Wilmington, DE), which is in contact with hot water (95–98 °C). The Mylar film creates a “window” that allows infrared radiation transmission, from the thermal energy of the water to the product, at wavelengths that corresponds with the absorption spectra of the water molecules in the solution or purée^24^. Radiation allows the product to dry quickly due to low resistance to the thermal conductivity of the film, which makes drying by RW an alternative for thermosensitive products such as probiotics. RW has been used successfully in mango^25^, pumpkin^26^, asparagus^27^, among others. Studies have reported that it retains color^28^, ascorbic acid, antioxidant activity^27^, carotenoids and capsaicinoids^29^ preserving the quality of the physicochemical properties as well as the bioactive compounds in food in a similar way to that obtained by lyophilization and better than that achieved by spray drying^25^.

Nevertheless, the potential of RW drying applied to encapsulation processes has been little explored. The encapsulation of orange oil by Refractance Window and spray drying was made by Cadwallader *et al*. (2010). Greater retention of orange oil was observed when using RW (75.7%) compared to spray drying (56.9%), and less formation of undesirable products such as “*limonene oxide*” was observed^30^. It is necessary to evaluate the potential of the Refractance Window drying as an encapsulation technology and to find the operational conditions in which probiotic survival and a low moisture content are achieved. For this purpose, mathematical modeling is a commonly used tool^31^. Modified Gompertz^32^, Buchanan^33^ and Whiting-Buchanan models^34^ have been used to predict microbial thermal inactivation in isothermal conditions. Thin-layer models such as Lewis^35^, Logaritmic^36^ and Midilli^37^, among others, have been used to study drying kinetics and to estimate the drying time of a product^31^.

Therefore, the purpose of this work was to study and mathematically model the survival of *Lactobacillus fermentum* K73 and drying kinetics with the use of two encapsulation matrices to select the process conditions that allows a high cell viability and a lower content of humidity, and thus, to explore the use of RW drying as a technology to encapsulate probiotics.

## Results

### Refractance Window drying as encapsulation technology and mathematical modelling of survival curves

**Figure 1:**
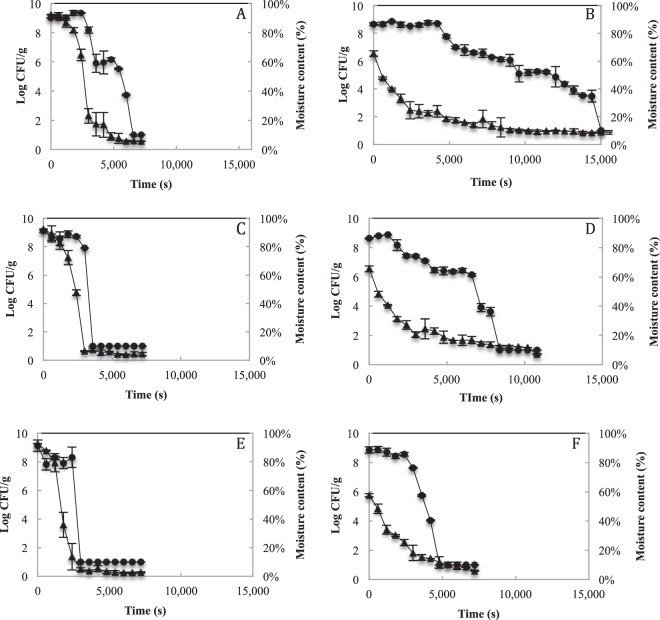
Refractance window drying of *Lactobacillus fermentum* K73 without carrier material (**A**,**C**,**E**) and with carrier material (**B,D,F**) at 333.15, 343.15 and 353.15 Kelvin.

Figure 1 shows the behavior of *Lactobacillus fermentum* K73 and the loss of moisture during the drying process by using RW at the three different temperatures evaluated: 333, 343 and 353 K. The culture medium as an encapsulating agent was evaluated (without carrier material). The viability of the microorganism was constant during the initial drying phase up to 2400 seconds for the three temperatures (Fig. 1A,C,E). Then, a rapid decrease in cell viability was observed at 343 and 353 K (Fig. 1C,E). During the drying process at 333 K, after the first 2400 s, there was a cellular decrease of 3 logarithmic units until 3600 seconds, and then an “intermittent lag phase” of 1200 s followed by a decrease in cell viability until 6600 seconds was observed (Fig. 1A).

The medium with an increase in solids of 32% with maltodextrin and whey (with carrier material) was evaluated. Results showed that at 333 K there was a change in the cellular concentration. The cellular concentration presented a “cyclic” behavior during the drying process. First, the cellular concentration decreased 1.7 logarithmic units from 4200 s to 5400 s; second, it had a minimum variation from 5400 s to 9000 s; third, the cellular concentration decreased 0.9 logarithmic units until 9600 s; fourth, the cellular concentration slowly decreased 1.62 logarithmic units until 14400 s of the drying process, finally, the minimum value of the cell count allowed by the sensitivity of the technique was recorded (1 Log CFU/g) at 15000 s (Fig. 1B). When the drying process was carried out at 343 K, it was observed that the cellular decrease was similar to that presented at a temperature of 333 K but with shorter cycles; between 6600 and 8400 s there is a decrease of 6.12 logarithmic units (Fig. 1D). Drying kinetics carried out at 353 K, showed that after 2400 seconds and until 4800 seconds a decrease of 8.55 logarithm units was observed (Fig. 1F).

Table 1 shows the values of the lag phase (*L*), the cell inactivation rate (*k*) and the statistical parameters used to evaluate the settings of the model. The Gompertz and Whiting & Buchanan models showed high values of R^2^ and adjusted R^2^ (R^2^ > 0.916–0.827, R^2^adj > 0.909–0.811) compared to the Buchanan model (R^2^ > 0.721, R^2^adj > 0.695). However, when the criteria “Sum of squares” and “Root mean squared error” were used to evaluate the settings of the model, it was observed that the Gompertz model presented the lowest values among the three evaluated models. Bias (Bf) and Accuracy factor (Af) were also used as quantitative indicators to measure the settings of the models. The Gompertz model and the Buchanan model showed results closer to 1 for bias and accuracy factor than the Whiting & Buchanan model.

**Table 1:** Kinetics parameters calculated by Gompertz, Buchanan, and Whiting and Buchanan models for the behavior of Lactobacillus fermentum K73 during Refractance Window drying and regression analysis.

Model	Temperature	Carrier material	Parameter	SS	RMSE	Bf	Af	R^2^	Adj R^2^
Gompertz model	333 K	With	k = 0.000596	1.907	0.595	1.039	1.137	0.934	0.931
			L = 4278.217						
		Without	k = 0.00216	0.411	0.988	1.024	1.196	0.916	0.909
			L = 2964.1						
	343 K	With	k = 0.00127	1.976	0.808	1.12	1.23	0.921	0.838
			L = 3249.2						
		Without	k = 0.016335	10.545	0.572	0.554	1.88	0.996	0.996
			L = 2937.045						
	353 K	With	k = 0.003608	15.754	3.176	0.799	2.105	0.984	0.982
			L = 2710.274						
		Without	k = 0.018283	0.024	0.769	1.006	1.056	0.966	0.957
			L = 2374.917						
Buchanan model	333 K	With	D = 2426.7	5.491	0.731	1.118	1.428	0.942	0.94
			k = 0.000949						
			L = 1745.5						
		Without	D = 902	0.817	1.151	1.044	1.172	0.861	0.848
			k = 0.002553						
			L = 1296.2						
	343 K	With	D = 1189.5	1.036	1.136	1.137	1.248	0.904	0.898
			k = 0.001936						
			L = 1467.2						
		Without	D = 667	2.242	1.934	0.986	1.267	0.769	0.748
			k = 0.003452						
			L = 924.8						
	353 K	With	D = 679.3	11.87	1.248	1.622	1.801	0.882	0.871
			k = 0.00339						
			L = 1050.8						
		Without	D = 535	0.782	2.398	1.014	1.179	0.721	0.695
			k = 0.004304						
			L = 400.6						
Whiting and Buchanan Model	333 K	With	F = 0.681749	0.057	0.189	0.98	1.144	0.989	0.988
			b = 0.0590093						
			L = 3151.0998						
			c = 0.000986						
		Without	F = 0.6065192	1	0.623	0.988	1.164	0.951	0.946
			b = 0.0046736						
			L = 2991.5256						
			c = 0.1928						
	343 K	With	F = 0.0000	4.165	0.851	0.822	1.452	0.827	0.811
			b = 0.011974						
			L = 2356.3677						
			c = 0.0021641						
		Without	F = 0.9957	0	0.085	1	1	0.999	0.999
			b = 0.0266						
			L = 2923.0074						
			c = 0.096638						
	353 K	With	F = 0.0000	5.711	0.275	0.091	11.314	0.99	0.989
			b = 0.0171974						
			L = 2720.4911						
			c = 0.0083191						
		Without	F = 0.9928	1032.908	0.672	0.999	1	0.976	0.971
			b = 0.0262						
			L = 2280.8472						
			c = 0.0966						

k = Inactivation rate (s^−1^), L = Lag phase (s), D = decimal reduction time (s), F = initial proportion in the less resistant fraction, b and c = model parameters, SS = Sum of squares, RSME = Root mean squared error, Bf = Bias factor, Af = Accuracy factor, R^2^ = R-squared, Adj R^2^ = Adjusted R-squared.

The samples without carrier material dryed at 343 K and 353 K obtained Bf values from 0.554 to 1.622 and Af values from 1.056 to 2.105 for the Gompertz and the Buchanan models. On the other hand, the Bf values were 0.091–1.0 and the Af velues 1–11.314 for the Whiting and Buchanan model on samples with carrier material dryed at 343 and 353 K. Bf and Af indicate a perfect “match” between the experimental and predicted data by the model when their values are 1^38^. When the Bf values are above 1 or below 1, the predicted values can be overestimated or underestimated^39^. Af must always be greater than or equal to 1, and the higher this value is the precision of the model is lost^38^. Consequently, the Gompertz model showed the closest values to 1 for Bf and Af in most of the evaluated conditions.

**Figure 2:**
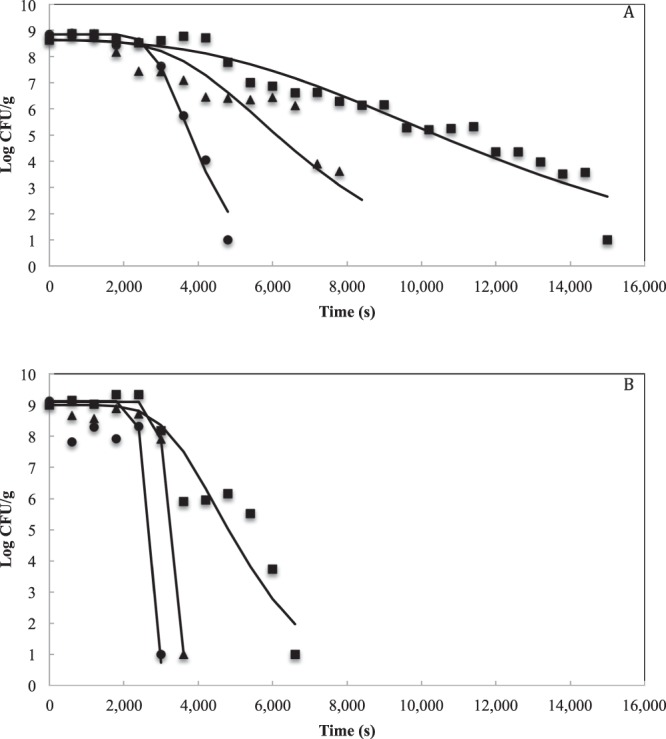
*Lactobacillus fermentum* K73 kinetics during Refractance Window drying with carrier material (**A**) and without carrier material (**B**) at 333.15 °K (■), 343.15 °K (◆) and 353.15 °K (●) using Gompertz Model. Comparison between experimental (symbols) and predicted (lines) values. N = cell density at any time, N_0_ = initial cell density.

According to the results obtained from SS, RSME, R^2^, R^2^adj, Bf and Af, the Gompertz model was selected to study the behavior of *L. fermentum* K73 during the RW drying process. Figure 2 shows the experimental data settings (symbols) *vs*. the predicted data (solid line) by the model. As the process temperature increases, the cellular inactivation rate (*k*) increases and the lag (*L*) phase decreases, which indicates the effect of temperature on the viability of the microorganism. In contrast, *k* is greater and *L* is lower when the culture medium is used as an encapsulating agent in comparison with the maltodextrin—whey matrix. The above shows the protective effect of the carrier materials during the drying process as stated above.

### Effect of temperature on the behavior of *Lactobacillus fermentum* K73

Table 2 shows the kinetic parameters, model parameters and the results of the linear regression analysis of the Gompertz-Arrhenius model. The lowest values of R^2^ and R^2^adj were observed when the model was applied to the drying kinetics of the microorganism (With carrier material: R^2^: 0.85, R^2^ adj: 0.848; Without carrier material: R^2^: 0.914, R^2^ adj: 0.912), which means that only 85% and 91% of the total variation can be explained by the model according to the type of carrier material. Additionally, the value obtained by Af was greater than 1 (Af = 1.319), which indicates that some experimental data differ from the predicted data (Liao *et al*.^34^). The lack of adjustment of the model can be explained by the difference of times of the lag phase, especially for the lowest assumed temperature (333 K). The lack of adjustment of this model, Gompertz-Arrhenius, was observed by Gil *et al*., when they were evaluating non-isothermal conditions with a slow heating treatment; they suggested improving the settings of the model by changing the sampling times, decreasing them in the initial phase and increasing them in the period of maximum inactivation rate^40^.

**Table 2:** Kinetics parameters and regression analysis results calculated by Gompertz-Arrhenius model, for the behavior of Lactobacillus fermentum K73 during Refractance Window drying process under non-isothermal conditions.

Carrier material	Temperature Dependent parameters		Model parameters	SS	RMSE	Bf	Af	R^2^	Adj R^2^
With	333 K	k = 0.203	a = 1553827.348	10.21	1.127	1.095	1.319	0.850	0.848
		L = 7612.7	b = 11346.132						
	343 K	k = 0.4545	c = 0.0005						
		L = 2821.68	d = 313						
	353 K	k = 0.8060							
		L = 1106.34							
Without	333 K	k = 0.0003	a = 47631.089	5.787	1.303	1.137	1.266	0.914	0.912
		L = 3979.48	b = 5295.4891						
	343 K	k = 0.1856	c = 0.002						
		L = 2504.12	d = 332.7						
	353 K	k = 0.711							
		L = 1617.62							

k = Inactivation rate (s^−1^), L = Lag phase (s), SS = Sum of squares, RSME = Root mean squared error, Bf = Bias factor, Af = Accuracy factor, R^2^ = R-squared, Adj R^2^ = Adjusted R-squared.

**Figure 3:**
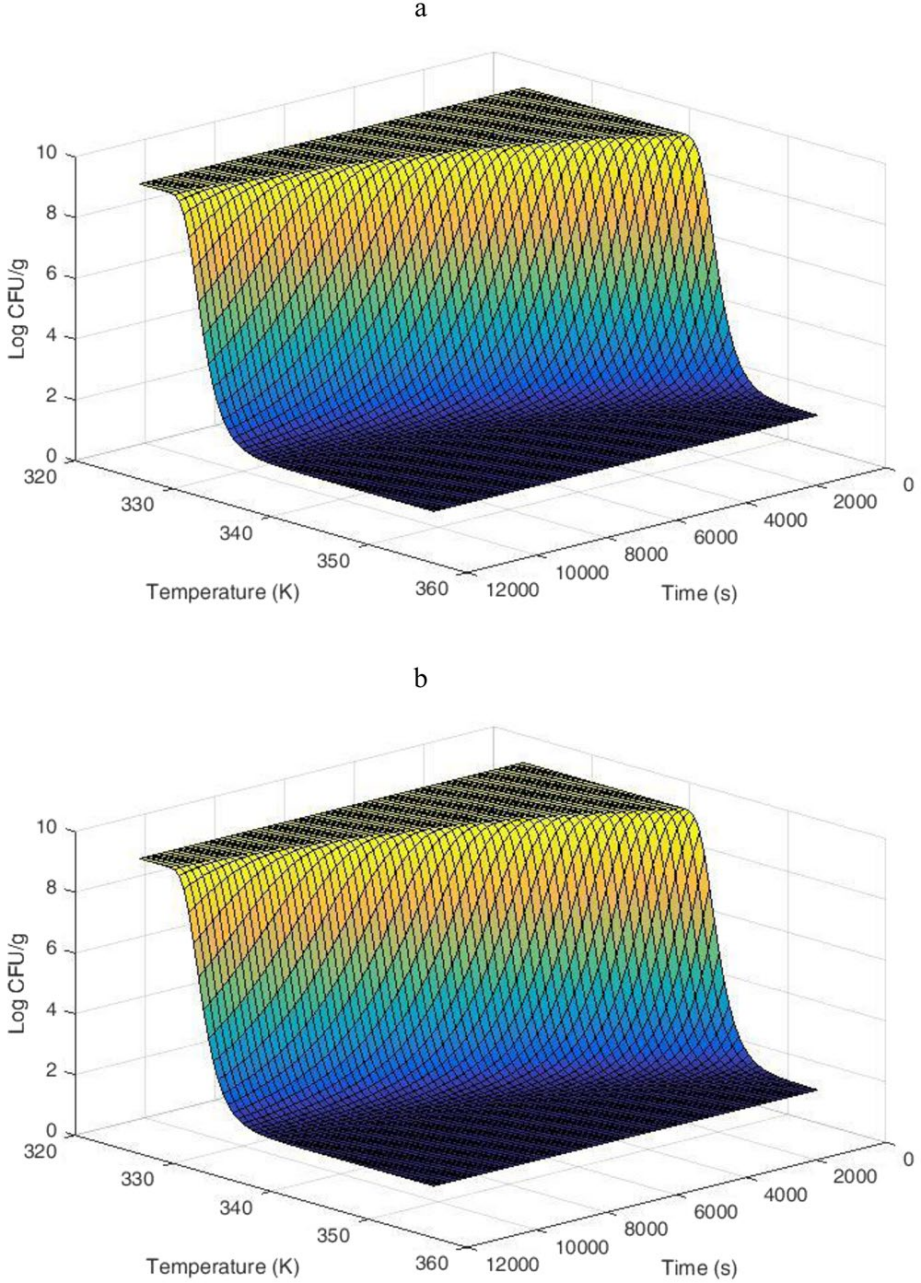
Effect of temperature on the behavior of *L. fermentum* K73 with (**a**) and without (**b**) carrier material, simulation of Gompertz-Arrhenius model (Eq. 7).

The values of the temperature-dependent parameters, *k* and *L* were calculated from equations 5 and 6 by using the values of the model parameters (Table 2). In contrast, Fig. 3 shows the effect of temperature on the behavior of *L. fermentum* K73 during the drying process.

### Thin-layer mathematical modelling

The humidity rate data obtained from the drying process at 333 K, 343 K and 353 K per RW of the matrices with and without carrier material were adjusted to eight thin-layer drying models. By means of the linear regression analysis, the parameters of each model and the statistical parameters (SS, RSME, R^2^ and R^2^adj) were determined and presented in Table 3. According to the results of the statistical parameters of all the thin-layer models for the evaluated conditions, the Midilli model showed the lowest values of SS (0.008–8.945) and RMSE (0.018–0.308), and the closest values to 1 for R^2^ (0.981–0.996) and R^2^Adj (0.978–0.994) (Table 2).

**Table 3:** Parameters and statistical results of thin-layer mathematical models for the moisture rate of the Refractance Window drying process.

Model and equation	Temperature	Carrier material	Parameter	SS	RMSE	R^2^	Adj R^2^
Lewis	333 K	With	*k*_*d*_ = 0.000397	20.485	0.811	0.682	0.670
MR = exp (−*k*_*d*_ t)^61^		Without	*k*_*d*_ = 0.0204	25.000	0.699	0.884	0.873
	343 K	With	*k*_*d*_ = 0.0224	6.011	0.056	0.975	0.973
		Without	*k*_*d*_ = 0.0252	48.199	0.142	0.879	0.868
	353 K	With	*k*_*d*_ = 0.0269	0.056	0.022	0.993	0.992
		Without	*k*_*d*_ = 0.0332	53.135	0.141	0.909	0.901
Page	333 K	With	*k*_*d*_ = 0.067	0.019	0.027	0.987	0.986
MR = exp (−*k*_*d*_ t^n^)^62^			n = 0.740				
		Without	*k*_*d*_ = 0.00257	23.458	0.727	0.899	0.889
			n = 1.32				
	343 K	With	*k*_*d*_ = 0.0746	0.169	0.018	0.992	0.992
			n = 0.7013				
		Without	*k*_*d*_ = 8.345E-08	0.018	0.027	0.993	0.992
			n = 4.3697				
	353 K	With	*k*_*d*_ = 0.0207	0.106	0.018	0.993	0.993
			n = 1.0684				
		Without	*k*_*d*_ = 0.00257	1.968	0.405	0.757	0.735
			n = 1.32				
Henderson & Pabis	333 K	With	a = 0.905	6.204	0.042	0.971	0.970
MR = a exp(−*k*_*d*_ t)^63^			*k*_*d*_ = 0.0213				
		Without	a = 1.2122	0.053	0.305	0.866	0.854
			*k*_*d*_ = 0.0241				
	343 K	With	a = 0.8958	2.915	0.041	0.966	0.964
			*k*_*d*_ = 0.0197				
		Without	a = 1.1973	43.453	0.110	0.867	0.854
			*k*_*d*_ = 0.029				
	353 K	With	a = 1.0188	0.053	0.018	0.993	0.992
			*k*_*d*_ = 0.0272				
		Without	a = 1.1657	4.633	0.128	0.903	0.894
			*k*_*d*_ = 0.0373				
Logaritmic	333 K	With	a = 0.9008	9.868	0.038	0.973	0.972
MR = a exp (−*k*_*d*_ t) + c^64^			*k*_*d*_ = 0.0246				
			c = 0.0318				
		Without	a = 1.8749	2.617	0.305	0.898	0.889
			*k*_*d*_ = 0.0101				
			c = −0.7257				
	343 K	With	a = 0.8659	0.391	0.029	0.982	0.981
			*k*_*d*_ = 0.0303				
			c = 0.1004				
		Without	a = 1.4758	13.000	0.099	0.886	0.875
			*k*_*d*_ = 0.0174				
			c = −0.321				
	353 K	With	a = 1.0242	0.063	0.018	0.993	0.992
			*k*_*d*_ = 0.0267				
			c = −0.0078				
		Without	a = 1.2645	8.848	0.120	0.910	0.902
			*k*_*d*_ = 0.0293				
			c = −0.1197				
Two terms exponential	333 K	With	a = 0.2463	3.225	0.032	0.985	0.984
MR = a exp (−*k*_*d*_ t) + (1 − a) exp (−*k*_*d*_ a t)^65^			*k*_*d*_ = 0.0755				
		Without	a = 2.4621	26.000	0.866	0.946	0.941
			*k*_*d*_ = 0.0381				
	343 K	With	a = 0.2481	1.615	0.029	0.987	0.986
			*k*_*d*_ = 0.0699				
		Without	a = 2.4602	25.012	0.074	0.940	0.935
			*k*_*d*_ = 0.0455				
	353 K	With	a = 1.4949	0.008	0.018	0.993	0.992
			*k*_*d*_ = 0.0317				
		Without	a = 2.5047	1.545	0.070	0.971	0.968
			*k*_*d*_ = 0.061				
Diffusion approximation	333 K	With	a = −0.0267	19.713	0.053	0.971	0.970
MR = a exp(−*k*_*d*_ t) + (1 − a)exp (−K b t)^66^			*k*_*d*_ = 0.0068				
			b = 3.0378				
		Without	a = 1.2767	40.000	0.866	0.889	0.879
			*k*_*d*_ = 0.0185				
			b = 0.9013				
	343 K	With	a = 0.5237	0.113	0.016	0.993	0.994
			*k*_*d*_ = 0.0121				
			b = 5.343				
		Without	a = 0	0.382	0.121	0.879	0.868
			*k*_*d*_ = 0.0219				
			b = 1.1534				
	353 K	With	a = 0	0.009	0.019	0.993	0.992
			*k*_*d*_ = 0.0226				
			b = 1.1859				
		Without	a = 0.000	0.238	0.309	0.909	0.901
			*k*_*d*_ = 0.02512				
			b = 1.3215				
Midilli	333 K	With	a = 0.999	0.019	0.027	0.987	0.986
MR = a exp(−*k*_*d*_ t^n^) + bt^67^			*k*_*d*_ = 0.0215				
			n = 0.741				
			b = 0.000				
		Without	a = 1.016	8.945	0.058	0.981	0.978
			*k*_*d*_ = 1.4169E-7				
			n = 3.141				
			b = 0.0001				
	343 K	With	a = 1.00519	0.166	0.018	0.992	0.992
			*k*_*d*_ = 0.067				
			n = 0.6978				
			b = 0				
		Without	a = 0.9715	0.017	0.026	0.992	0.991
			*k*_*d*_ = 5.33E-06				
			n = 4.2102				
			b = 4.1595E-14				
	353 K	With	a = 1.0069	0.008	0.018	0.993	0.993
			*k*_*d*_ = 0.07609				
			n = 1.0598				
			b = 0.000				
		Without	a = 0.997	0.070	0.308	0.996	0.994
			*k*_*d*_ = 7.144E-06				
			n = 3.4433				
			b = 0.000107				
Verma	333 K	With	a = 0.604	0.017	0.025	0.989	0.988
MR = a exp (−*k*_*d* 0_ t) + (1 − a) exp (−*k*_*d* 1_ t)^68^			*k*_*d* 0_ = 0.0149				
			*k*_*d* 1_ = 0.0789				
		Without	a = 4.823	1.075	0.866	0.878	0.867
			*k*_*d* 0_ = 0.00295				
			*k*_*d* 1 = _0.0016				
	343 K	With	a = 0	43.971	0.536	0.775	0.761
			*k*_*d* 0_ = 0.00295				
			*k*_*d* 1_ = 0.0016				
		Without	a = 8.0139	0.283	0.104	0.885	0.874
			*k*_*d* 0_ = 0.0094				
			*k*_*d* 1_ = 0.008007				
	353 K	With	a = 1.9784	0.008	0.018	0.993	0.992
			*k*_*d* 0_ = 0.0212				
			*k*_*d* 1_ = 0.0168				
		Without	a = 14.6764	0.859	0.309	0.911	0.903
			*k*_*d* 0_ = 0.01654				
			*k*_*d* 1_ = 0.01570				
			*k*_*d* 2_ = 0.037				

SS = Sum of squares, RSME = Root mean squared error, R^2^ = R-squared, Adj R^2^ = Adjusted R-squared.

**Figure 4:**
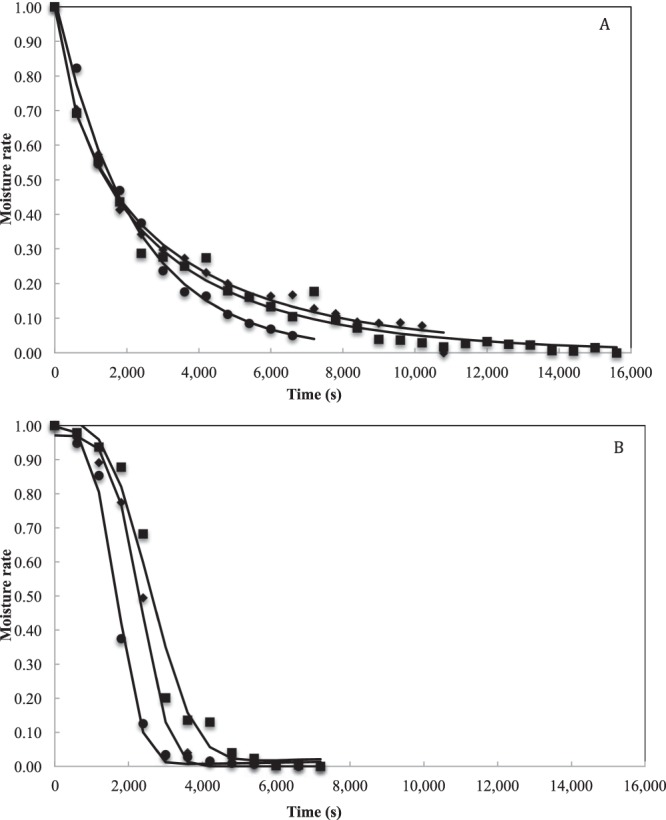
Thin layer Refractance Window drying curves of *L. fermentum* K73 with (**A**) and without (**B**) carrier material. Comparison between experimental (symbols) and predicted (lines) values of moisture ratio using the Midilli model for 333.15 K (■), 343.15 K (◆) and 353.15 K (●).

Figure 4 shows the evolution of the humidity rate as a function of the drying time at the three evaluated temperatures, with and without carrier material. The drying time to reach balanced moisture content was 13800 s, 9000 s and 7200 s at 333, 343 and 353 K for samples with carrier material (Fig. 4A); and for samples without carrier material 6000 s, 5400 s and 4800 s at 333, 343 and 353 K, respectively (Fig. 4B). In both types of samples, as the temperature of the water bath increases, the drying process is accelerated, decreasing the residence time of the mixture on the Mylar film sheet^41^. Moreover, it is observed an increase in drying times of 2.3 times, 1.66 times and 1.50 times at 333, 343 and 353 K, respectively when using the mixture with maltodextrin and whey (with carrier material) compared to the culture medium as carrier material, showing the effect of the solids concentration of the mixtures regarding the time and the drying rate.

The equation describing the Midilli model is shown in Table 3. Where *k*_*d*_ is the constant of the drying rate (s^−1^), *t* is the time (s), and *a, n* and *b* are the shape parameters of the model. The value of the drying constant, *k*, is greater in the mixture with carrier material than in the mixture without carrier material, and increases with higher water bath temperature (Table 3), which is consistent with the drying kinetics (Fig. 4).

## Discussion

The present work studied the RW drying technology as an alternative for the encapsulation process of probiotics with promising results using the survival matematically models and thin layer models to find the optimal conditions. Survival of the microorganism *Lactobacillus fermentum K73* after the drying process is essential to obtain a product able to be used as a probiotic. The probiotic potential of *the Lactobacillus fermentum K73* has been demonstrated by Cueto *et al*.^42^ In addition, this microorganism has a hypocholesterolemic effect to adsorb cholesterol on its cell membrane and for the activity of the bile salt hydrolase enzyme^11^

The survival curves of the *L. fermentum* K73 had a cyclic behavior. This type of “cyclic” behavior that occurred in the kinetics of drying with and without carrier material is called “intermittent lag phase”, and has been reported by different authors when studying non-isothermal conditions during cell growth^43–45^. The intermittent lag phase is related to an immediate adjustment of the microbial population to a new processing temperature^45^. Zotarelli *et al*.^23^ demonstrated, through a thermographic study, that a temperature gradient in the food matrix occurs when dried by RW at 368 K (water bath temperature). When placing the food matrix on the Mylar film, the temperature of the product rises to 346.35 K in the first 300 s of drying and to 361.65 K after 900 s of the drying process^23^.

Zotarelli *et al*.^23^ showed a drying process with similar conditions as the reported on this study (12°Brix, food matrix thickness of the 3 mm, and Mylar film thickness of the 0.33 mm) with a difference in the composition of the products to be dried. Consequently, if it is considered that during the drying process there is a gradual increase in temperature, the following four events could be generated in co-occurrence or in a combination of them. First, during the drying process at 333 K the increase in temperature in the encapsulation matrix could be slower than at 343 and 353 K, allowing the microorganism to adapt for short periods of time to the increase in temperature, generating the intermittent lag phase^46,47^. Second, the encapsulation matrix with carrier material had a protective effect during the drying process independently of the processing temperature. The hydrophilic groups of the denatured whey proteins from the culture medium (sterilized at 394 K for 900 s) could interact with the other components of the carrier material (maltodextrin and whey) resulting in the formation of a “gel-like” thin layer, which protects the microorganism from the migration of intracellular water to the environment^48,49^. Third, during the RW drying, the energy transfer is carried out by conduction, radiation and, to a lesser extent, convection^50^. The energy transfer by conduction increases the temperature in the sample (Zotarelli *et al*.^23^) and it could generate an increase in the cell inactivation rate (Table 2-Gompertz Model), decreasing the time of the initial lag phase (Table 2-Gompertz Model) and loss of moisture content in the sample (Figs 1 and 4). In contrast, the transfer of energy by radiation from the plastic to the Mylar film is easier, when the refraction between film-sample interfaces is reduced (sample with high moisture content, 92.4 ± 0.001% (wet basis)), cell viability decreases rapidly, especially in samples without carrier material^24^. Finally, cell death can be generated by an increased in the destabilization of the cell membrane, induction of “lyotropic membrane transition” from a crystalline liquid phase to a gel phase (permeabilization of the cell membrane) and increased contact with the oxygen molecules in the environment, creating reactive oxygen species at intracellular level^51,52^.

The Gompertz, Buchanan and Whiting & Buchanan models were used to study the behavior of the microorganism during the drying process. The Gomperzt-Arrhenius model (Equation 7) presented in this study corresponds to the derivation and simplification of the model developed by Gil *et al*.^40^, which has been reported to describe microbial inactivation as a function of temperature and time^40^. The model was applied under the assumption that cell growth or regeneration does not occur during the drying process. Results showed that by increasing the temperature and drying time, cell viability decreased. It was also observed that the addition of carrier materials favored the survival of the microorganism within the range of the evaluated temperatures. These results being consistent with the analysis of the Gompertz model under isothermal conditions, aforementioned. On the other hand, when increasing the temperature, there was an increase in the cell inactivation rate (*k*) and a decrease in the lag phase (*L*) independent of the use or not of the carrier material. However, when no carrier material was used, the rapid increase in *k* and the decrease in *L* generated a steep cell survival curve that resembles a straight line. This correlation of the kinetic parameters with the shape of the curve was similar to that reported by Corradini *et al*. (2007) who modeled heat inactivation under non-isothermal conditions for *Salmonella enteritidis*^47^.

The thin layer models were evalued to study the drying kinetics. The Midilli model was selected as the suitable thin-layer model to predict the characteristics of the drying process of the mixtures with and without carrier material in the encapsulation process of *Lactobacillus fermentum* K73 through RW. The aforementioned shows the effect of the solids concentration on the drying process. The total solids of the culture medium (8%) increased with maltodextrin and whey (40%), decreasing the initial moisture content and prolonging the drying times^53^.

According to the drying kinetics, it can be observed that the difference in drying is more noticeable during the initial stages of the process when the greater amount of water in the product is removed. During the drying process, the interaction between the denatured whey (from the culture medium) with the maltodextrin and the whey (present in the samples with carrier material), form a pseudo-viscous layer that obstructs the migration of water from inside the sample with carrier material to the surrounding environment, which increases the drying times. In addition, it is observed how the increase in temperature favors the migration of water from the product by transferring heat from the circulating water^54^, the aforementioned is observed in the increase of *k*_d_ as temperature increases. The use of the modified Midilli and Gompertz model allowed observing that there is a correlation between the drying rate (*k*_d_) and the cellular inactivation rate (*k*). When the drying temperature increases, *k*_d_ and *k* increase, which indicates that the faster the moisture in the product is lost, the faster the cell death rate increases in the product. The above is consistent with the phenomena aforementioned, where it is stated that cell death can be caused by the migration of water from the microorganism to the environment.

The selection of the drying condition that favored the encapsulation process was defined under the following criteria: (i) 6 Log CFU/g as the minimum cell concentration required for the consumption of a probiotic product (Pan *et al*. 2014) and (ii) moisture content less than 10%. The modified Gompertz model was used to calculate the time in which 6 Log CFU/g was obtained in both encapsulation matrices, then, the Midilli model was used to calculate the humidity at that time. Results showed that when using the culture medium without carrier material a moisture content between 3.5% and 9.3% is acquired compared to the matrix with carrier material where the moisture content was between 11.9–16.3% for the three processing temperatures. Therefore, the use of the medium as an encapsulation matrix allows obtaining a product with a moisture content of less than 10%. In contrast, the Midilli model was used to determine the time in which the moisture content was 10% in the matrix without carrier material, and then the cell concentration was calculated with the Gompertz modified model at that specific time. Results showed that at a drying temperature of 353 K, it is obtained 9.1 Log CFU/g with a moisture percentage of 10% at 2460 s of drying time, this being the condition that simultaneously meets the proposed criteria.

Finally, the viability of the microorganism was affected by the increase in drying temperature, the drying time and the encapsulation matrix. It was possible to determine the moment in which the concentration of the microorganism is ideal for obtaining a probiotic product with low moisture content (≤10% (wet basis)) and without the addition of carrier materials, which is represented in the low costs of preparation and processing of the product. The modified Gompertz model and the Gompertz-Arrhenius model were used as a tool to study the behavior of the microorganism during the drying process. The models were consistent in predicting that the cell inactivation rate increases and the Lag phase decreases as the drying temperature increases, independently of the use or not of carrier material. In contrast, the use of the Midilli model allowed to model the drying kinetics of both types of products; the drying and the cellular inactivation constant, respectively, showed to have a relation regarding the moisture loss speed with respect to the cell death speed. The process parameters determined for a successful encapsulation process were: 353 K, without carrier material and 2460 seconds of drying time. To this condition a product with 9.1 Log CFU/g and moisture content of 10% is obtained. With this result, it is proved that the Refractance Window drying can be a technically viable technology for the encapsulation of probiotics.

## Methods

### Materials

Agar and broth MRS (Man, Rogosa, Sharpe) and peptone water were obtained from Sharlau Microbiology (Barcelona, Spain). Yeast extract and maltodextrin were purchased from Oxoid Limited (England) and Shandong WNN Industrial Company Ltd (Shandong, China), respectively. Sweet whey was composed by: protein 11.67% (w/w), lipids 2.0% (w/w), carbohydrates 51.64% (w/w), ashes 10.9% (w/w) and was acquired from a local diary company (Cundinamarca, Colombia).

### Strain and culture conditions

*Lactobacillus fermentum* K73 (GenBank KP784433) was isolated from *suero costeño* (typical Colombian food) and characterized as a potential probiotic^11,42^. The strain was stored at −80 °C with 20% sterilized glycerol as crioprotectant in MRS broth^42^. *L. fermentum* K73 was propagated two times in MRS broth at 37 °C for 24 hours before the experiment was carried out.

The culture medium was prepared with 8% sweet whey and 0.22% yeast, and it was adjusted to pH 5.5 and sterilized at 121 °C for 15 min. The fermentation process was carried out in 1 L bioreactor with a workload of 800 mL at 37 °C and agitation at 100 rpm for 10 h. *L. fermentum* K73 was inoculated at 10% (v/v). The cell count was done after the fermentation process as shown in section *Microbiological analysis*.

### Encapsulation matrices

The drying process was carried out with the culture medium with and without carrier material.

#### With carrier material

Powder mixture of maltodextrin and sweet whey (0.6:0.4) was hydrated with the culture medium with grown microorganisms. The final solids concentration was 40%. The carrier material was fixed according to the reported by Aragon-Rojas *et al*.^55^, where an optimization of the carrier material content was developed. The mixture was homogenized with magnetic stirrer during 30 minutes at 130 rpm. Cell count was done as described in section *Microbiological analysis* after the homogenization process. The final cell count was 8.99 ± 0.145 Log CFU/g.

#### Without carrier material

The culture medium was dried with the purpose to evaluate the potential as carrier material. The final solids concentration was 8% and the final cell count was 9.19 ± 0.203 Log CFU/g.

### Refractance Window drying

A Laboratory-scale Refractance Window dryer was used with the same principle as industrial equipment^24,56^. The RW consists of a tray (0.9 m by 0.6 m) with the Mylar film (D type, DuPont, USA) at the top. Hot water (333 K, 343 K, 353 K) comes from the thermostatic bath and circulates to the container. All samples were placed on the Mylar film as a thin layer of 3 mm. Samples were taken each 10 minutes for microbiological analysis (section *Microbiological analysis*) and moisture content determination (section *Moisture content determination*) until the drying process was completed.

### Microbiological analysis

The plate count method was used to determine the number of viable probiotic. Serial 1:9 dilution in peptone water (0.1% w/v) and spread plating on MRS agar were performed^57^. The first dilution was homogenized by using a vortex during 10 min. The samples were incubated at 37 °C, during 24 h in aerobic conditions^11^. The result was expressed as colony forming units (CFU) per gram.

### Moisture content determination

The initial moisture content and the moisture loss from samples during drying were determined by oven method at 105 °C until constant moisture^58^. Approximately 1 g was taken from the Mylar film with a spatula for each sample extraction.

### Mathematical modelling of survival curves

Three models were chosen to evaluate the behavior of *Lactobacillus fermentum* K73 during the drying process: Gompertz, Buchanan, and Whiting and Buchanan model.

*Gompertz model* is used to describe log-linear kinetics as well as those containing shoulder and/or tailing effects^32,40^. The model is described by the equation 1 (Eq. 1):

1 $$LogN=Log{N}_{0}-Log\frac{{N}_{0}}{{N}_{f}}\ast e\,(-e((\frac{ke(1)}{Log(\frac{{N}_{0}}{{N}_{f}})})(L-t)+1))$$

Where *N* represents the cell density at time (*t*) in seconds, *N*_0_ and *N*_*f*_ are the initial and final cell density respectively, *k* the maximum inactivation rate constant and *L* is the parameter time (the shoulder).

*Buchanan model* is used to fit the data in a log-linear function with the presence of a shoulder as a lag time before to start the decline cell death^59^, the equation (Eq. 2) is the following:

2 $$Log\frac{N(t)}{{N}_{0}}=-\,(\frac{t-L}{D})$$

Where *N(t)* is the cell density at time (*t*, seconds), *N*_0_ is the initial cell density, *t* is the time in seconds, *L* is the duration of lag period prior to initiation of inactivation (seconds), and D is the D value or decimal reduction time^59^. *D value* was used to find *k* (death rate constant): *k* = ln(10)/D^33^.

*Whiting and Buchanan model* fit the sigmoidal curves with or without shoulder and tail, so it can be used to fit six different kinds of microbiological behavior^33^. Equation 3 describes the model:

3 $$Log\frac{{N}_{t}}{{N}_{0}}=Log(\frac{F(1+{e}^{-bL})}{1+{e}^{b(t-L)}}+\frac{(1-F)(1+{e}^{-cL})}{1+{e}^{c(t-L)}})$$

Where *N*_*t*_ is the cell density at time (*t*, seconds), *N*_0_ is the initial cell density, *t* is the time in seconds, *L* is the duration of lag period prior to initiation of inactivation (seconds), *F* is the initial proportion in the less resistant fraction, (1 − *F*) is the more resistant fraction, *b* is the inactivation rate of the major population group and *c* is the inactivation rate of the minor population group.

### Effect of temperature on the behavior of *Lactobacillus fermentum* K73

The modified Gompertz model^40^ was selected to describe the behavior of the microorganism in function of time varying temperature (Eq. 4).

4 $$LogN=Log{N}_{0}{\int }_{0}^{1}\,[k\,\exp (1)exp\,\,(\frac{kexp(1)}{Log(\frac{{N}_{0}}{{N}_{f}})}(L-t^{\prime} )+1)exp(-\exp (\frac{k\,\exp (1)}{Log(\frac{{N}_{0}}{{N}_{f}})}(L-t^{\prime} )+1))]dt^{\prime} $$

*N*_0_ and *N*_*f*_ represent the initial and final cell density respectively, *L* is the time parameter of the duration of lag period (seconds) and *k* is the maximum inactivation rate constant. The parameters *L* and *k* are dependent of the temperature. The Arrhenius – Type equation (Eq. 5) describes the effect of the temperature on *L*:

5 $$L=a\,\exp (b(\frac{1}{T}-\frac{1}{{T}_{ref}}))$$

*T* is the temperature in Kelvin, *T*_*ref*_ is a fixed reference temperature, and *a* and *b* are parameters of the model.

Equation 6 is used to relate the inactivation rate constant with the temperature^59^, where *c* and *d* are parameters and *T* is the temperature in Kelvin:

6 $$k=c{(T-d)}^{2}$$

The integration of equations 4, 5 and 6, result in a mathematical model (Gompertz and Arrhenius model) that allows describing the amount of the cell density through time in three different temperatures (Eq. 7):

7 $$\begin{array}{rcl}Log\,N & = & Log{N}_{0}-(((c{(T-d)}^{2}\,\exp (1))/((c{(T-d)}^{2}\,\exp (1)/\mathrm{Log}(\frac{{N}_{0}}{{N}_{f}})))\\  &  & \times \exp (\,-\,\exp ({\rm{c}}(({\rm{a}}\,\exp ({\rm{b}}(\frac{1}{{\rm{T}}}-\frac{1}{{{\rm{T}}}_{ref}})))-{\rm{t}})+1))\\  &  & +(((({\rm{c}}{({\rm{T}}-{\rm{d}})}^{2}\,\exp (1)/((c\,{(T-d)}^{2}\\  &  & \exp (1)/Log(\frac{{N}_{0}}{{N}_{f}})))\,\exp \,(\,-\,\exp (c(a\,\exp (b(\frac{1}{T}-\frac{1}{{{\rm{T}}}_{ref}})))+1)))\end{array}$$

### Mathematical modelling of thin-layer drying

The drying curves obtained were fitted with eight different thin-layer drying models shown in Table 3. The moisture ratio (MR) in these model equations (Eq. 8) were defined as follows:

8 $$MR=\frac{M-Me}{Mo-Me}$$

Where M, Mo and Me were instantaneous, initial and equilibrium moisture contents, respectively.

### Statistical analysis

The experimental data from Refractance Windows experiments (Microbiological analysis and Moisture content determination) were fitted in the mathematical models showed in Table 1 and Table 2. The nonlinear regression analysis was conducted by SAS software version 2.0.4 (SAS Institute, Inc., Cary, North Carolina). Bias factor (Bf) (Eq. 9) and Accuracy factor (Af) (Eq. 10) were used to fit the mathematical models from Table 1 ^60^.

9 $$Bf={10}^{{\sum }^{}\frac{Log(\frac{predicted}{observed})}{n}}$$

10 $$Af={10}^{({\sum }^{}\frac{|Log(\frac{predicted}{observed})|}{n})}$$

Sum of squares (SS), Root mean squared error (RSME), R-squared (R^2^), R-squared adjusted (R^2^adj) were used as criteria to assess the goodness-of-fit of microbial and thin-layer drying kinetic models to experimental data.
